# Analysis of Potential Genes, Oxidative, Metabolic, and Hormonal Markers Associated with Postpartum Disorder Susceptibility in Barki Sheep (*Ovis aries*)

**DOI:** 10.3390/vetsci12030219

**Published:** 2025-03-02

**Authors:** Asmaa Darwish, Ali J. Mohamed, Salah H. Faraj, Ahmed El-Sayed, Mansour A. Alghamdi, Ahmed M. Sallam, Attia Eissa, Belal F. Farag, Yasser Kamel, Eman M. Embaby, Ahmed Ateya

**Affiliations:** 1 Department of Animal Health and Poultry, Animal and Poultry Production Division, Desert Research Center (DRC), Cairo 11753, Egypt; asmaa_vet25@drc.gov.eg; 2 Department of Animal Production, College of Agriculture, University of Misan, Maysan 62001, Iraq; ali.jasim@uomisan.edu.iq; 3 Department of Biology, College of Science, University of Misan, Maysan 62001, Iraq; salah81ss@uomisan.edu.iq; 4 Department of Anatomy, College of Medicine, King Khalid University, Abha 62529, Saudi Arabia; m.alghamdi@kku.edu.sa; 5 Genomics and Personalized Medicine Unit, The Center for Medical and Health Research, King Khalid University, Abha 62529, Saudi Arabia; 6 Animal and Poultry Production Division, Department of Animal and Poultry Breeding, Desert Research Center, Cairo 11753, Egypt; sallam@drc.gov.eg; 7 Department of Animal Medicine (Internal Medicine), Faculty of Veterinary Medicine, Arish University, Arish 45511, Egypt; attia.ahmed@vet.aru.edu.eg; 8 Animal Production Department, Faculty of Agriculture, Al-Azhar University, Assiut Branch 71524, Egypt; BelalMohammed.4419@azhar.edu.eg; 9 Department of Microbiology, Faculty of Medicine, Rabigh, King Abdulaziz University, Jeddah 21589, Saudi Arabia; ymahmod@kau.edu.sa; 10 Department of Microbiology, Desert Research Center, Cairo 11562, Egypt; 11 Department of Physiology, Faculty of Veterinary Medicine, Mansoura University, Mansoura 35516, Egypt; emanmohamed@mans.edu.eg; 12 Department of Development of Animal Wealth, Faculty of Veterinary Medicine, Mansoura University, Mansoura 35516, Egypt

**Keywords:** Barki sheep, gene expression, postpartum disorders, APPs, hormones

## Abstract

This study purpose was to determine the gene expression as well as serum profile of acute phase proteins (APPs) and hormonal indicators linked to Barki sheep’s susceptibility to postpartum issues. Three equal-sized groups (each with fifty ewes) were created from the blood of 150 adult Barki ewes: the control group (CG), the inflammatory postpartum disorders group (IPG), and the non-inflammatory postpartum disorders group (NIPG). The expression levels of the oxidative stress (*PGC-1α*, *SIRT1*, *GCLC*, *GCLM*, and *EPAS1*) and metabolic (*FBXL12*, *KPNA7*, and *LRRK1*) genes were significantly higher in postpartum disorders sheep than in resistant ones. Ewes with inflammatory postpartum illnesses showed significantly higher levels of the examined markers than did the non-inflammatory and control groups. The serum profile analysis also revealed that the levels of Fb, Cp, Hp, SAA, cortisol, TIBC, UIBC, and ferritin were significantly higher in the IPG than in the NIPG and CG. Serum insulin, iron, transferrin, and Tf Sat.% levels, however, were all markedly lower. On the basis of the variance in the genes being studied and the modulation in the serum indicators being studied, it should be possible to monitor the health status in postpartum problems of sheep.

## Simple Summary

The postpartum period represents one of the most challenging times for Barki ewes, marked by reduced feed intake, shifts in hormonal and metabolic status, and physiological stresses, many of which are immunological in nature. In a study involving 150 adult ewes, researchers divided the animals into three groups after initial blood sampling: a control group, a group with non-inflammatory postpartum disorders, and a group with inflammatory postpartum disorders, each consisting of 50 ewes. Results revealed significantly elevated expression of metabolic and oxidative stress-related genes in ewes affected by postpartum complications compared to those showing natural resistance. Additionally, distinct profiles emerged across the groups in blood levels of some acute phase proteins and hormonal markers. The obtained findings suggest that a significant impact and relatedness of the investigated indicators on postpartum disorders in sheep.

## 1. Introduction

The Barki sheep, named after Libya’s Barka province, is native to Egypt’s scenic northwestern coast. This resilient breed plays an essential role in the livelihoods of the local communities, blending tradition and sustainability in one of the region’s most iconic agricultural practices [1]. This breed comprises approximately 470,000 individuals, accounting for 11% of the total sheep population in Egypt, and is distributed across a broad geographic area, spanning from eastern Libya to the western region of Alexandria, Egypt. Known for its resilience, the Barki breed demonstrates remarkable tolerance to harsh environmental conditions, including high temperatures, limited forage availability, and heat stress, underscoring its importance to the region [2]. Basic data on the body conformation and productivity of Barki ewes have been documented [3].

The postpartum period in ewes, lasting 45 days after parturition, is recognized as a critical phase marked by physiological stress due to reduced feed intake and significant hormonal and metabolic adjustments associated with both parturition and lactation. This period is often accompanied by immune-related physiological stressors [4]. If these physiological shifts are not managed effectively, ewes may develop a range of inflammatory and non-inflammatory conditions, including mastitis, endometritis, and hypocalcemia [5,6]. Such conditions negatively impact flock productivity, as they contribute to decreased reproductive rates, shorter productive lifespans in ewes, and elevated mortality rates among ewes and lambs, ultimately affecting economic outcomes for breeders [6].

Blood biochemical analyses provide extensive insight into an animal’s nutritional status, health, and overall well-being, making them a valuable tool for assessing general health [7]. Deviations in specific blood parameters from established normal ranges can aid in the differential diagnosis of diseases, offering details on the severity of infection and the degree of tissue damage extent [8].

Acute phase proteins (APPs) are a group of some proteins, released by the hepatocytes into blood, which play crucial roles in minimizing pathological damage, restoring homeostasis, and restricting microbial growth in infected animals without relying on antibodies [9]. The levels of APPs in the blood are modulated by a range of physiological and pathological factors, such as age, sex, diet, pregnancy, lactation, and environmental conditions [10]. These proteins can be either positive or negative APPs, relying on whether their blood concentrations rise or fall in response to infection, inflammation, or other internal and external stressors. These fluctuations offer important diagnostic and prognostic insights, with significant interspecies variability in response patterns [11,12].

In ruminants, haptoglobin (Hp) serves as the primary acute phase protein, while serum amyloid A (SAA) is another positive APP that typically increases during the acute phase response [13].

Through the improvement of animal health, advanced molecular genetic techniques provide promising supplemental approaches for disease control [14]. Numerous genetic techniques, particularly single nucleotide polymorphisms (SNPs), have been approved with great success and have been linked to livestock resistance and susceptibility to various diseases [15,16]. These results highlight the differences in disease susceptibility and resilience across host genomes [17].

Currently, however, limited information exists on the relationship between acute phase proteins (APPs), hormonal shifts, and gene expression associated with postpartum pathological disorders in sheep. Furthermore, there is a lack of effective diagnostic and related biomarker tools for these conditions, which constrains progress in developing preventive and therapeutic measures. This study aims to address these gaps by examining gene expression patterns and serum profiles of APPs, hormonal and iron profile markers in Barki sheep, with a focus on postpartum disorders. Through this approach, the research seeks to provide critical insights into the underlying molecular mechanisms to monitor these conditions, supporting more effective management strategies for ovine health.

## 2. Material and Methods

### 2.1. Animals

This work utilized one hundred fifty mature Barki ewes with body weights ranging from 45.8 to 52.2 kg (48 ± 2.2 kg) and an average age of 3 to 4 years (3.5 ± 0.3 years). Conducted at Egypt’s Sustainable Development Center for Matrouh Resources (SDCMR), part of the Desert Research Center (DRC), the study followed rigorous clinical assessments based on standardized protocols, ensuring thorough documentation of all findings. The ewes stayed in clean, semi-open, well-ventilated, shaded pens and received a controlled daily mash of 1 kg of concentrate feed mixture (CFM) and 600 g of alfalfa hay per head, with unrestricted access to fresh water. The CFM formulation included wheat bran (300 kg), soybeans (250 kg), corn (400 kg), premix (1 kg), calcium carbonate (20 kg), sodium chloride (10 kg), and Fylax (0.5 kg). All animal handling and procedures were approved by the DRC Animal Health Ethics Committee, with reference number AH/NO2023, and were conducted in agreement with relevant guidelines and ARRIVE regulations, prioritizing ethical standards throughout the study [18].

### 2.2. Study Design

The ewes in this study were categorized into three groups based on their postpartum health status, each group containing fifty ewes. The first group, designated as the control group (CG), consisted of clinically healthy ewes (experienced normal parturition and rapid postpartum recovery, displaying typical appetite, stable body temperature, no abnormal uterine secretion, and healthy udders).

The second group represented animals with inflammatory disorders during postpartum period (IPG) and was comprised of twenty-five ewes diagnosed with endometritis (characterized by fever, persistent foul-smelling uterine discharge, anorexia, and depression) and twenty-five ewes had mastitis (evidenced by hyperthermia, decreased feed consumption, red enlarged swollen and sensitive udders, abnormal milk appearance, foul odor, clots in milk, and pain during milking due to teat injury and cracks).

The third group members had non-inflammatory disorders during postpartum (NIPG). Among these, ten individuals encountered dystocia (typically during their first birth with a larger lamb), ten showed symptoms of hypocalcemia (displaying recumbency, muscle tremors, and rapid recovery post calcium supplementation), ten experienced uterine prolapse, ten had retained placenta, and ten went through abortion. This categorization provided a comprehensive look into the spectrum of postpartum health challenges faced by Barki ewes, each with distinct clinical markers and impacts on animal well-being.

### 2.3. Blood Sampling and Measurements

In all groups, ten ml of fresh blood was extracted via jugular vein puncture from each ewe, then divided into three parts. EDTA and heparin calcium 5000 I.U. were added to the first and second part, respectively, to interfere with the coagulation cascade. The EDTA blood was utilized for real time PCR while the second and third parts were centrifuged at 3000 r.p.m. for 20 min at 37 °C, to obtain plasma and serum, respectively. Plasma and serum samples were stored at −80 °C in clean Eppendorf tubes for further APPs, iron profile, and hormonal analysis.

### 2.4. Total RNA Extraction, Reverse Transcription, and Quantitative Real-Time PCR

Total RNA was extracted from sheep blood using the Trizol reagent (RNeasy Mini Ki, Catalogue no. 74104, Tustin, CA, USA) as stated in the manufacturer’s instructions. Quantifying and qualifying the amount of isolated RNA was carried out using the NanoDrop^®^ ND-1000 Spectrophotometer. cDNA was produced for each sample in compliance with the manufacturing procedure (Thermo Fisher, Catalog no. EP0441, London, UK). The coding fragments of genes encoding metabolic (*FBXL12*, *KPNA7*, and *LRRK1*) and oxidative stress (*PGC-1α*, *SIRT1*, *GCLC*, *GCLM*, and *EPAS1*) genes were evaluated using quantitative RT-PCR with SYBR Green PCR Master Mix (2× SensiFAST^TM^ SYBR, Bioline, CAT No: Bio-98002, London, UK).

Using real-time PCR, the relative amount of mRNA was determined using the SYBR Green PCR Master Mix (QuantiTect SYBR green PCR kit, Catalogue no. 204141, Germantown, MD, USA). Primers were developed using the published sequence of *Ovis aries* from PubMed as a basis (Table 1). To act as a constant reference for normalization, the housekeeping gene GAPDH was used. The reaction mixture had the following composition: 0.5 µL of each primer, 8.25 µL of RNase-free water, 0.25 µL of reverse transcriptase, 3 µL of total RNA, 4 µL of 5× Trans Amp buffer (London, UK), and 12.5 µL of 2× QuantiTect SYBR green PCR master mix (Intron, Bremen, Germany).

A thermal cycler was used to run the following program on the resultant reaction mixture: 30 min at 50 °C for reverse transcription, 10 min at 94 °C for primary denaturation, 40 cycles at 94 °C for 15 s, 1 min at the annealing temperature as indicated in Table 1, and 30 s at 72 °C. A melting curve analysis was carried out to verify the PCR product’s specificity at the conclusion of the amplification process. According to the 2^−ΔΔCt^ method, the relative expression of each gene in each sample was calculated and compared to the *GAPDH* gene [19].

### 2.5. APPs, Iron Profile, and Hormonal Analysis

To evaluate various plasma and serum markers, a range of assays and kits were employed. Plasma fibrinogen (Fb) concentrations, along with serum SAA and Hp, were measured by ELISA method using IBL International Crop (Toronto, ON, Canada)^®^ kits. Serum caeruloplasmin (Cp) levels were determined using a turbidimetric method with Elabscience kits (Houston, TX, USA)^®^. For hormonal assays, cortisol and insulin concentrations were assessed through chemiluminescence immunoassay (CLIA) using Diasorin kits (Diasorin, Saluggia, Italy)^®^.

Serum iron (SI) and total iron-binding capacity (TIBC) were measured spectrophotometrically with kits from Biodiagnostic Company^®^ (Montevideo, Uruguay). Serum ferritin levels were obtained through the CLIA method using Abnova kits (Taipei, Taiwan)^®^, while transferrin (Tf) levels were analyzed via a turbidimetric method with Elabscience kits (Houston, TX, USA)^®^.

Transferrin saturation (Tf sat. %) = SI/TIBC × 100

Unsaturated iron-binding capacity (UIBC) = TIBC − SI

### 2.6. Statistical Analysis

Statistical parameters were presented as mean ± standard deviation (SD), and data analysis was performed using SPSS version 23. A one-way ANOVA test was applied to compare group means, followed by Tukey’s HSD test for post hoc analysis to pinpoint specific group differences, with significance set at *p* < 0.05.

For estimating cut-off points, sensitivity, specificity, and likelihood ratios (LRs) of the APPs across the IPG as well as the NIPG compared to the CG, Graph Pad Prism version 8 was used.

Chi-square tests assessed the distribution of identified SNPs in genes between resistant ewes and affected ones, while Pearson correlation determined relationships between APPs, iron profile, hormonal parameters, and gene expression of the tested enzymes, with correlation coefficient (r) and *p*-values reported.

The other values were calculated as follows:

PPV% = True positives/Total positives × 100

NPV% = True negatives/Total negatives × 100

Accuracy rate% = True positives + True negatives/Total population × 100

Percentage of increase % = Mean concentration in IPG or NIPG − Mean concentration in CG/Mean concentration in CG × 100

## 3. Results

### 3.1. Patterns for Transcript Levels of Assessed Indicators

**Figure 1:**
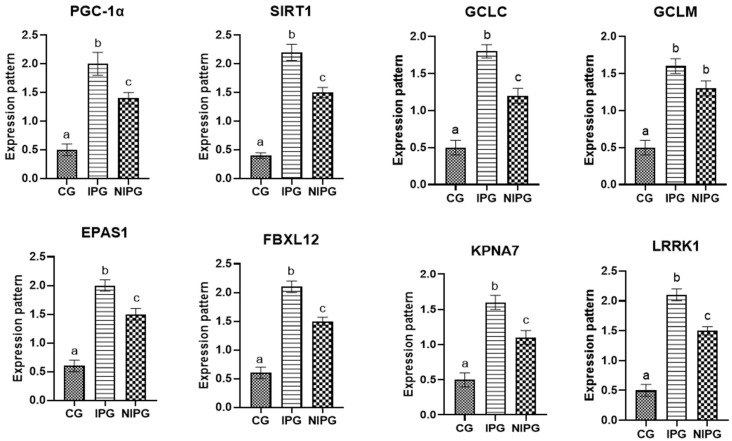
Relative expression patterns of oxidative stress and metabolic genes in the healthy group (CG), inflammatory postpartum group (IPG), and non-inflammatory postpartum group (NIPG). Results are expressed as means ± SEM. a, b, c small alphabetic letters show significance when *p* < 0.05.

The transcript profiles for the evaluated indicators are shown in Figure 1. For the genes *PGC-1α*, *SIRT1*, *GCLC*, *GCLM*, *EPAS1*, *FBXL12*, *KPNA7*, and *LRRK1*, the expression levels of these genes were significantly greater in postpartum-disordered sheep than in resistant ones. Ewes with inflammatory postpartum illnesses showed significantly higher levels of the examined markers than did the non-inflammatory and control groups.

### 3.2. APPs, Iron Profile, and Hormonal Markers

Table 2 summarizes the iron profile, hormone markers, and APP serum concentrations in the CG, IPG, and NIPG. Serum levels of APPs (Fb, Cp, Hp, SAA), cortisol, TIBC, UIBC, and ferritin were significantly (*p* < 0.05) higher in the IPG and NIPG than in the CG, and in the IPG than in the NIPG. In contrast, there was a significant (*p* < 0.05) drop in serum levels of insulin, serum iron, transferrin, and Tf Sat. % in the IPG and NIPG when compared to the CG and the IPG when compared to the NIPG, respectively (Table 2). Regarding the diagnostic utility of the estimated APPs markers in the postpartum disorders, Table 3 shows that the IPG and NIPG had 100% sensitivity and NPV for all measured APP markers, whereas Fb and Cp had low LRs (less than 5) and Hp and SAA had moderate LRs (5–10). In contrast, Hp experienced the largest percentage increase in both the IPG and NIPG, followed by SAA.

### 3.3. Correlation Between Gene Expression Pattern and Serum Profile of Acute Phase Proteins and Biochemical Markers in IPG and NIPG

**Figure 2:**
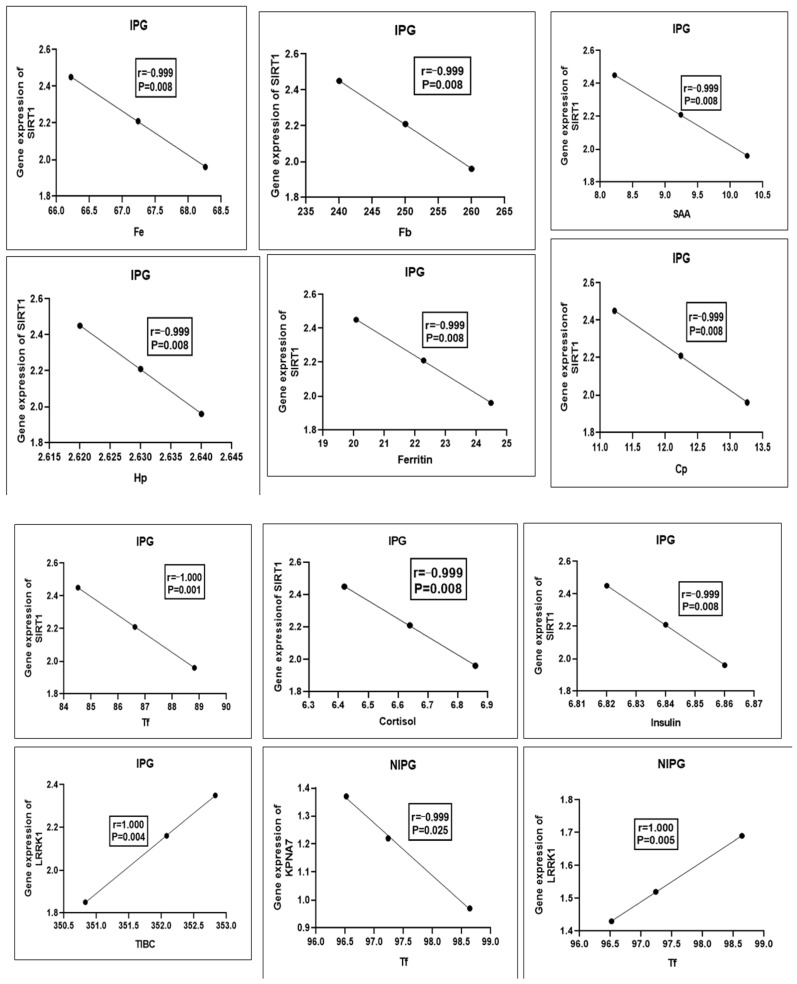
The correlation between the genetic parameters and biochemical parameters in the inflammatory postpartum group (IPG) and non-inflammatory postpartum group (NIPG) (Pearson’s correlation test), considered significant when *p* < 0.05.

Figure 2 shows the relationship between the gene expression patterns in the IPG and NIPG and the serum profiles of acute phase proteins and biochemical markers. While there was a negative correlation (r = −1, *p* = 0.01) between the mRNA levels of SIRT1 in the IPG and the blood levels of iron, Fb, Hp, SAA, Cp, cortisol, insulin, ferritin, and Tf, there was a positive association (r = 1, *p* = 0.007) between the blood levels of TIBC and the mRNA levels of *LRRK1*. In the NIPG, serum Tf levels showed a positive correlation with *LRRK1* mRNA levels (r = 1, *p* = 0.007) and a negative correlation with *KPNA7* mRNA levels (r = −1, *p* = 0.01).

## 4. Discussion

A crucial time in the life of Barki ewes, the postpartum stage is linked to many immunological and endocrine changes. The majority of these changes start in the prepartum phase, gradually intensify until they reach their peak after parturition, and continue to be noticeable in the early postpartum phase before gradually decreasing until normalcy [20]. Regrettably, these changes typically lead to the formation of either inflammatory or non-inflammatory postpartum illnesses. According to [21], postpartum diseases in Barki ewes provide a significant challenge to animal husbandry as they may lead to elevated mortality rates and reduced rates of fertility in the ewes that were impacted. Our goal was to look for any possible links between APPs, hormonal and iron profile changes, and gene expression and postpartum disorders in sheep.

We looked at how the metabolic condition and oxidative stress changed in ewes with postpartum problems as opposed to healthy ones. The transcript profiles for the evaluated markers, which were determined by analyzing the mRNA levels of *PGC-1α*, *KPNA7*, *SIRT1*, *GCLM*, *GCLC*, *FBXL12*, *EPAS1,* and *LRRK1,* were significantly greater in postpartum disorders sheep than in resistant sheep. Ewes with inflammatory postpartum illnesses showed significantly higher levels of the examined markers than did the non-inflammatory and control groups. This study is the first investigation of the transcript levels of markers linked to sheep postpartum issues.

Ref. [22] suggested that SIRT1 has a regulatory role in the oxidative stress response of cow mammary cells. The authors found that the SIRT1 gene and SIRT1 protein were more highly expressed after being exposed to H_2_O_2_. Additionally, it boosted the phosphorylation of AMPK, increased the gene expression of CAT, GCLM, GCLC, PGC-1α, SOD2, and NQO1, increased the protein expression of PGC-1α, Nrf2, NQO1, and HO-1, and decreased the phosphorylation of NF-κB. The authors of [23] assessed the relationship between *EPAS1* mutations and congestive heart failure in cows. It has previously been documented how immunological and antioxidant markers of postpartum illnesses are expressed in animals. For example, Ref. [24] showed that sheep with postpartum issues had significantly greater gene expression profiles of IL1-ß, TNF-α, IL5, IL6, TLR4, and Tollip than did resistant ewes. In contrast, SOD and CAT genes showed the reverse trend.

A2M, IRAK3, TLR2, FCAMR, CCl2, iNOS, KCNT2, ADAMTS20, MAP3K4, FKBP5, MAPK14, and EPHA4 gene expression levels were significantly greater in buffaloes with endometritis than in resistant buffaloes, per [25]. Buffaloes with endometritis showed significantly decreased levels of expression of the NDUFS5, RXFP1, TGF-β, CAT, SOD3, and GPX transcription factors. IL-8, IL-17, LR4, NFKB, NCF4, SLCA11A1, HMOX1, ST1P1, Keap1, OXSR1, and SERP1 were significantly higher in the buffaloes with endometritis, according to [26]. Conversely, there was a down-regulation of the genes encoding SOD, CAT, Nrf2, NDUFS6, and PRDX2. When comparing endometritis-affected cows to resistant ones, while the expression of IL10, ATOX1, and GST was much lower, that of TNF-α, TLR4, TLR7, NCF4, OXSR1, LITAF, TKT, RPIA, and AMPD1 was significantly increased [27]. In buffaloes with inflammatory reproductive disorders, the expression of the inflammatory genes (LGALS, IKBKG, IL1B, RANTES, CCL2, HMGB1, MASP2, and S-LZ) was significantly higher [28].

Research has demonstrated that SIRT1 and AMPK can interact [29]. By deacetylating AMPK, Sirt1 can boost its phosphorylation; conversely, AMPK can control the NAD+/NADPH ratio, which in turn increases SIRT1 activity. By deacetylation of PGC-1α, increased SIRT1 activity enhances mitochondrial oxidative dephosphorization [30]. SIRT1 activation and AMPK phosphorylation also increase Nrf2 expression [31], which in turn stimulates HO-1 [32] and NQO1 [33] expression. By increasing the expression of antioxidant enzymes like CAT, GSH, and SOD [34], this interaction increases the antioxidant capacity of cells. Important antioxidant markers that also show oxidative stress in breast tissue are the GCLC and GCLM [35]. Angus cattle with pulmonary hypertension have higher levels of the endothelial PAS domain-containing protein 1 gene (*EPAS1*), which codes for a hypoxia-inducible factor 2 alpha (HIF2α) twofold variant with serine (S) at position 610 and threonine (T) at position 606 [36]. It was suggested that this HIF2α T606/S610 mutation exhibited a prominent gain-of-function activity [37].

The 40-amino-acid F-box motif (*FBXL12*) is a characteristic of the F-box protein family, which includes proteins like F-box and leucine-rich repeat protein and F-box and WD repeat domain containing 9 (FBXW9). Protein-ubiquitin ligases are functioned by F-box proteins [38]. F-box proteins interact with ubiquitination targets via other protein interaction domains [38]. Karyopherin subunit 7 (*KPNA7*) has a role in the import of nuclear proteins and aids in the construction of the mitotic spindle, which guides the chromosomes to be duplicated during mitosis, both of which increase the precision of cell division [39]. Although it has little effect on the characteristics of bone formation, leucine-rich repeat kinase 1 (*LRRK1*) is crucial for regulating osteoclast activity, cytoskeletal architecture, and bone resorption [40]. In the mouse spleen, LRRK2 protein is expressed by both B cells and macrophages, and B2 cells, a subset of B cells, express LRRK2 mRNA noticeably higher than B1 cells [41]. These results imply that LRRK2 plays significant functions in the immune system. Compared to resistant kids, diarrheal goat kids had considerably higher levels of *KPNA7*, *FBXL12*, and *LRRK1* in their gene expression profiles [27].

The significant increase in the expression pattern of oxidative stress metabolic markers in ewes with inflammatory postpartum illnesses may be due to the release of pro-inflammatory cytokines and cytotoxic radicals by the phagocytic cells [42]. Furthermore, reactive nitrogen intermediates are important radicals that play a complicated function in the inflammatory process [43]. By promoting lipid peroxidation and DNA breakage, excessive ROM production harms preimplantation embryos and frequently leads to embryonic mortality [44]. Additionally, it hinders the development of embryos in non-inflammatory postpartum conditions. Moreover, oxidative stress during pregnancy has been connected to cases of dystocia [45]. Similarly, postpartum metabolic problems are associated with oxidative stress, which increases the inflammatory response [46,47].

A strong acute phase response was found in both the NIPG and IPG in the current study. This was demonstrated by the higher than expected concentrations of APPs in the IPG and NIPG when compared to the CG. Acute phase proteins are excellent indicators of several diseases in humans and animals. They play a vital role in the host’s innate immunological reaction. They are non-specifically released by hepatocytes as a reply to pro-inflammatory cytokine stimuli, which limit the growth of microorganisms and prevent them from spreading until the establishment of particular immunity. APP blood levels aid in the assessment of the disease’s stage and the monitoring of treatment plans [21,48,49,50,51]. The acute phase response is normally expected in the peri- and postpartum stage in ruminants. It participates in proper parturition and fetal membrane evacuation at the postpartum stage. Previous research has extensively documented the elevation of APPs in a variety of inflammatory and non-inflammatory postpartum disorders in various ruminants [21,48,49,50,51]. They mainly attributed this elevation to the negative energy balance and hyperketonemia in this stage, which stimulate the pro-inflammatory cytokines and subsequent APPs release [50]. The infection and inflammation are additional causes for APR magnification in the IPG (compared to the CG and NIPG) in this work [52], while the hypocalcemia in the NIPG group is a more specific cause for APR in this group, as calcium is an deeply incorporated in cell proliferation, differentiation, and motility, muscle contraction, hormone secretion, glycogen metabolism second messenger, and as enzyme cofactor. The hypocalcemia induced the cortisol and pro-inflammatory cytokines concentration, causing an acute phase response in the liver [48]. Abortion for non-infectious causes in cow stimulate also APP release from the liver, especially SAA [49]. Retained placenta, uterine prolapse, and dystocia in cow were associated with a marked increase in SAA and Hp blood levels [53,54].

Regarding the hormonal alterations, both the IPG and NIPG showed a large drop in serum insulin concentration and a significant increase in cortisol levels when compared to the CG. These results were consistent with earlier research [55,56] that found similar endocrine changes in postpartum illnesses in ruminants. Due to the hypoglycemia caused by the previously described immunological response and milk production, the post-partum stage is typically linked to a negative energy balance and hyperketonemia [24,56,57]. Consequently, the pancreatic islet decreased its insulin secretion and the adrenal cortex activated to enhance cortisol secretion, resulting in the observed hypoinsulinemia and hypercortisolemia in the IPG and NIPG. Hence, this induces a hyperglycemic condition by decreasing the absorption of glucose by various bodily tissues, augmenting the processes of gluconeogenesis and glycogenolysis in the liver and kidneys, and inducing lipolysis of fat reserves to preserve the energy required for survival. Additionally, hypercortisolemia reduces energy expenditure by stimulating the pituitary gland to secrete growth hormone and by obstructing the thyroid gland from secreting T3 and T4. Furthermore, these hormonal alterations in the IPG and NIPG were also influenced by the pain and stress associated with inflammatory (mastitis and endometritis) and non-inflammatory postpartum illnesses (retained placenta, hypocalcemia, dystocia, uterine prolapse, abortion) [55,56].

The IPG and NIPG’s iron profiles revealed a marked hypoferremia, which was followed by a discernible rise in TIBC and UIBC and a decrease in Tf sat.%. However, in both afflicted groups, hypoferremia was found as a result of bleeding during parturition, anorexia, and decreased feed intake associated with the disease’s etiology [58,59]. The hypoferremia observed in this study’s IPG and NIPG was accompanied by hyperferritinemia and hypotransferrinemia, which are common findings during infections. These are caused by activated pro-inflammatory cytokines, which also promote the release of hepcidin, which inhibits intestinal iron absorption and suppresses transferrin activity, preventing the invasive microorganisms from obtaining the iron they need for growth [6].

Although this mechanism is mostly preventive, it eventually causes the hypoferremia in sick animals to increase, leading to severe anemia and a dismal prognosis for the affected instances. In this case of hyperferritinemia and hypotransferrinemia, documented acute phase response in the IPG and NIPG may also disintegrate. In contrast, transferrin is a negative acute phase reactant that down-regulates during infection, and ferritin is a positive acute phase reactant that up-regulates during infection [5,60]. Previous research on postpartum problems showed similar alterations in their iron profiles [58]. It is important to note that inflammatory postpartum disorders (IPG) exhibit more acute phase response and altered hormonal and iron profiles than non-inflammatory postpartum disorders (NIPG) due to the existence of an infectious agent and extensive damaged tissue [52].

The tested APPs showed high sensitivities, specificities, PPVs, NPVs, and ARs, according to the results in Table 3, while LR and percentage of increase introduced Hp followed by SAA as the best markers among the estimated markers. The findings corroborated those of other researchers who suggested using APPs as diagnostic markers for both non-inflammatory and inflammatory postpartum disorders in ruminants, particularly Hp and SAA [21,51].

## 5. Conclusions

Significant gene expression, hormonal, and biochemical changes linked to postpartum issues in Barki sheep are convincingly demonstrated by the results. The mRNA levels of oxidative stress and metabolic markers were significantly different between postpartum disorders and in resistant sheep. The varying patterns of expression and biochemical profile in these investigated markers could be linked with postpartum disorders and used to track the health of sheep.

## Figures and Tables

**Table 1:** Oligonucleotide primer sequences, accession numbers, annealing temperatures and PCR product sizes of immune and antioxidant genes used in real-time PCR.

Examined Marker	Primer	Product Size (bp)	Annealing Temperature (°C)	GenBank Isolate	Origin
*PGC-1α*	F5′-CCGTGCTCAGAGCTTCTCAA-3 R5′-CTGCTGTTCCGGTTCTCTGT-3′	80	58	AY575608.1	Present Research
*SIRT1*	F5′-TGCTCGCCTTGCAATAGACT-3′ R5′-TCCACTGCACAGGCACATAC-3′	213	60	XM_027962451	
*GCLC*	F5′-CATTTGCAAAGGTGGCAACG-3′ R5′-TGGTGTCGATGGACATGAGC-3′	101	60	XM_015102644.4	
*GCLM*	F5′-AGTCACCAGCCTATCTGGTAT-3′ R5′-AGGACTGAGCAGGCCATATC-3′	248	60	XM_060411958.1	
*EPAS1*	F5′-′ CGACCATGAGGAGATCCGTG-3′ R5′-GGGCAGTTGCTGTAGACCTT-3′	84	58	JF267354.1	
*FBXL12*	F5′-TACACGGTATGCGGGTCTG-3′ R5′-CATGACTTTGGGCCGCATCT-3′	199	58	XM_042249906.2	
*KPNA7*	F5′-ATCCAGCAGCTAATCGCCTG-3′ R5′-GTGAAGTTAGCCACCGTCCA-3′	107	60	XM_015104257.4	
*LRRK1*	F5′-GTGAAGGCAGGGTTGCAGAAC-3′ R5′-ATCCTCACTGCCGTTTCACAA-3′	224	58	XM_027956999.3	
*GAPDH*	F5′-TGGTGAAGGTCGGAGTGAAC-3′ R5′-CCGTTCTCTGCCTTGACTGT-3′	187	60	NM_001190390.1	

**Table 2:** APPs, cortisol, insulin, and iron profile of the studied groups.

Parameter	CG	IPG	NIPG
Fb (mg/dL)	144.75 ± 4.1 a	268.50 ± 19.8 b	241.10 ± 32.6 b,c
Cp (mg/dL)	6.63 ± 1.1 a	13.26 ± 1.4 b	10.11 ± 0.07 b,c
Hp (g/dL)	0.65 ± 0.02 a	2.67 ± 0.04 b	1.90 ± 0.04 b,c
SAA (mg/L)	2.83 ± 0.02 a	9.51 ± 1 b	7.22 ± 0.67 a,b
Cortisol (μg/dL)	3.50 ± 0.1 a	6.75 ± 0.2 b	5.55 ± 0.5 b,c
Insulin (μIU/mL)	7.41 ± 0.1 a	6.89 ± 0.05 b	7.05 ± 0.01 b,c
Serum iron (μg/dL)	96.93 ± 0.3 a	67.55 ± 1.1 b	80.83 ± 1.5 b,c
TIBC (μg/dL)	338.04 ± 2.9 a	352.38 ± 1.5 b	345.13 ± 2.2 b,c
UIBC (μg/dL)	241.11 ± 2.8 a	284.83 ± 1.5 b	264.30 ± 2.5 b,c
Transferrin(mg/dL)	136.16 ± 2.7 a	89.55 ± 3.2 b	98.50 ± 1.4 b,c
Tf sat. %	28.68 ± 0.2 a	19.17 ± 0.3 b	23.42 ± 0.4 b,c
Ferritin (ng/mL)	11.58 ± 0.3 a	22.94 ± 2.4 b	17.11 ± 0.6 b,c

a (significant with CG), b (significant with IPG), c (significant between the three groups), considered statistically significant at *p* < 0.05.

**Table 3:** ROC curve analysis for the measured APPs in the IPG and NIPG compared to the CG.

Parameters	Fb (mg/dL)		Cp (mg/dL)		Hp (g/dL)		SAA (mg/L)	
	IPG	NIPG	IPG	NIPG	IPG	NIPG	IPG	NIPG
Cut off	147.5	147.5	7.60	7.60	0.68	0.68	2.84	2.84
Sensitivity%	100	100	100	100	100	100	100	100
Specificity%	70	70	75	75	90	90	80	80
LR	3.33	3.33	4	4	10	10	5	5
PPV%	76.92	76.92	80	80	90.91	90.91	83.33	83.33
NPV%	100	100	100	100	100	100	100	100
Accuracy rate%	85	85	87.5	87.5	95	95	90	90
% of increase or decrease	85.49	66.56	100	52.49	310.77	192.30	236.04	155.12

## Data Availability

Upon reasonable request, the appropriate author will share supporting data for the study’s conclusions.

## Abbreviations

Cp	Caeruloplasmin
Fb	Fibrinogen
*FBXL12*	F-Box and Leucine-Rich Repeat Protein 12
*GCLC*	Glutamate-cysteine ligase
*GCLM*	Glutamate-Cysteine Ligase Modifier Subunit
Hp	Haptoglobin
*KPNA7*	Karyopherin Subunit Alpha 7
*LRRK1*	Leucine-rich repeat kinase 1
*PGC-1α*	Peroxisome proliferator-activated receptor gamma coactivator 1-alpha
SAA	Serum amyloid A
SI	Serum iron
*SIRT1*	Sirtuin 1
TF sat. %	Transferrin saturation
TIBC	Total iron-binding capacity
UIBC	Unsaturated iron-binding capacity
